# Factorial variation of saccade vigor with dual decision processes

**DOI:** 10.1167/jov.26.11.2

**Published:** 2026-11-04

**Authors:** Wanyi Lyu, Harry Parmar, Thomas R. Reppert, Martin Paré, Jeffrey D. Schall

**Affiliations:** 1 Department of Biology, York University, Toronto, ON, Canada; 2 Centre for Vision Research, York University, Toronto, ON, Canada; 3 Centre for Integrative and Applied Neuroscience, York University, Toronto, ON, Canada; 4 Department of Biomedical and Molecular Sciences, Queen's University, Kingston, ON, Canada; 5 Department of Psychology, The University of the South, Sewanee, TN, USA; 6 Centre for Neuroscience, Queen's University, Kingston, ON, Canada

**Keywords:** movement vigor, visual search, stochastic accumulator model, evidence accumulation, selective influence

## Abstract

Canonical stochastic models of decision-making treats decision and action as independent and sequential processes; however, studies involving limb movements consistently show that movement duration and kinematics are influenced by the quality of evidence. We examined the influence of evidence accumulation processes on eye-movement kinematics by testing whether saccade velocity varies with the quality of evidence in macaque monkeys performing a visual search Go/NoGo task. The elongation of a color singleton cued the Go/NoGo stimulus–response rule, while its location specified the saccade target. We factorially manipulated the efficiency of stimulus–response cue discrimination by varying the elongation of the singleton and the efficiency of singleton localization by varying the color similarity between the singleton and distractors. The effectiveness of the manipulations was revealed by the response times on correct trials that were separately modified by the singleton localizability and stimulus–response cue discriminability, by the incidence of localization and response selection errors with separately modified error response times. Saccade vigor (peak velocity normalized for amplitude) was higher on correct relative to error trials and was inversely proportional to response time. Saccade vigor was separately modified by singleton localizability and stimulus–response cue discriminability, controlling for variation of pupil diameter. Distinct patterns of error rates and saccade vigor across monkeys indicated individual differences in decision-making strategies. These findings demonstrate that the process selecting endpoints can influence both the timing and dynamics of saccadic eye movements. Incorporating saccade vigor can provide constraints on biologically plausible decision models and help address the persistent challenge of model mimicry.

## Introduction

Sequential sampling accumulator models explain variation of response time (RT) in terms of the dynamics of evidence accumulation to a threshold (Forstmann, Ratcliff, & Wagenmakers, 2016; Ratcliff, 1978; Ratcliff, Smith, Brown, & McKoon, 2016). How the response is executed is beyond the scope of these models, but recent research measuring RTs with manual responses has reported systematic variation in response speed or force as well as in RTs (Gallivan, Chapman, Wolpert, & Flanagan, 2018; Molano-Mazón et al., 2024; Servant, Logan, Gajdos, & Evans, 2021; Servant, White, Montagnini, & Burle, 2015; Wispinski, Gallivan, & Chapman, 2020). Hence, new accumulator models have been devised to incorporate the variation in execution dynamics (Dendauw et al., 2024; Molano-Mazón et al., 2024).

Whereas a given manual movement can be executed with some variation in speed and force, saccadic eye movements are supposed to be executed with strict co-variation of velocity, amplitude, and duration (e.g., Bahill, Clark, & Stark, 1975; cf. Quaia, Paré, Wurtz, & Optican, 2000). This main sequence relationship among the peak velocity, amplitude, and duration can optimize the balance between accuracy and duration of the eye movement (Harris & Wolpert, 2006). Yet, multiple researchers have shown that saccade velocity can vary systematically with motivation (Reppert, Lempert, Glimcher, & Shadmehr, 2015; Shadmehr, Reppert, Summerside, Yoon, & Ahmed, 2019; Takikawa, Kawagoe, Itoh, Nakahara, & Hikosaka, 2002), speed–accuracy trade-off (Reppert, Servant, Heitz, & Schall, 2018b), and urgency (Seideman, Stanford, & Salinas, 2018; Thura, 2020). Generally, saccades are faster when subjects are more motivated and when the expected value or probability of reward is high (Haith, Reppert, & Shadmehr, 2012; Schütz & Stewart, 2025; Shadmehr, 2010).

The purpose of this study was to determine whether the evidence supporting visual search singleton localization and stimulus–response discrimination influenced the timing and vigor of saccadic responses. We quantified saccade vigor in a double factorial visual search task designed to separately manipulate two evidence accumulation processes (Lowe, Reppert, & Schall, 2019). Previously, we showed that the systematic variation of response times across the 2 × 2 conditions could be explained as the outcome of two, separate evidence accumulation processes (Lowe et al., 2019). Because saccade peak velocity scales with amplitude (Bahill et al., 1975), comparisons of movement speed across trials require normalization for amplitude. Here, we computed saccade vigor, defined as peak velocity normalized for saccade amplitude (e.g., Reppert et al., 2015), which has been interpreted as proportional to energy expenditure (Shadmehr et al., 2019; Spering, 2022).

For two reasons, we also measured pupil diameter. First, pupil dynamics have been linked to cognitive processes and attention load (Beatty, 1982; Joshi & Gold, 2020; Kahneman, 1973; Van Der Wel & Van Steenbergen, 2018; Van Slooten, Jahfari, Knapen, & Theeuwes, 2018; Wang & Munoz, 2015). Second, pupil dynamics can influence gaze measures with video-based eye-tracking system (Nyström, Hooge, & Andersson, 2016). To determine whether observed variations in saccade velocity were confounded by pupil size, we assessed whether pupil diameter at saccade initiation covaried with saccade vigor and modulated any effects of singleton localizability and stimulus–response cue discriminability.

## Methods

### Data acquisition

This study complied with the ARVO Statement for the Use of Animals in Ophthalmic and Vision Research. All procedures were approved by the Vanderbilt Institutional Animal Care and Use Committee in accordance with the U.S. Department of Agriculture and Public Health Service Policy on Humane Care and Use of Laboratory Animals.

Two macaques (*Macaca radiata*), identified as Da (male, 12.8 years old at the time of study, 8.0 kg) and Le (male, 7.5 years old, 13.1 kg), performed a Go/NoGo visual search task. The macaque monkeys earned rewards for shifting their gaze to an elongated color singleton or withholding their gaze shift if the singleton was square. The shape of the stimulus–response cue was varied from high to low discriminability relative to the square NoGo cue. Interleaved, the chromatic similarity of the singleton and distractors was varied from high to low localizability. Moreover, the hues assigned to the singleton and the distractors were randomized across trials. The high and low efficiency of stimulus–response cue discrimination and the high and low efficiency of singleton localization created a 2 × 2 factorial design.

Eye position and pupil diameter (1000 Hz) were sampled with video-oculography (EyeLink; SR Research, Ottawa, ON, Canada). The monkeys earned fluid rewards for shifting gaze to a color singleton presented with seven distractor items in a circular array, unless the stimuli were squares (Figure 1A). Trials began with fixation of a central stimulus. After a minimum interval (random non-aging delay between 800 and 2000 ms), eight iso-eccentric, equiluminant, square or rectangular stimuli were presented at 6° or 8° eccentricity. The singleton and distractors were square on ∼25% of trials, cuing a NoGo response, and the monkeys were rewarded for maintaining fixation at the central spot for 1000 ± 0 ms. If stimuli were elongated, then the monkeys were rewarded for shifting their gaze to the singleton and maintaining fixation for 800 ± 0 ms (Le) or 1000 ± 0 ms (Da). The intertrial interval was fixed at 2 seconds.

**Figure 1.**
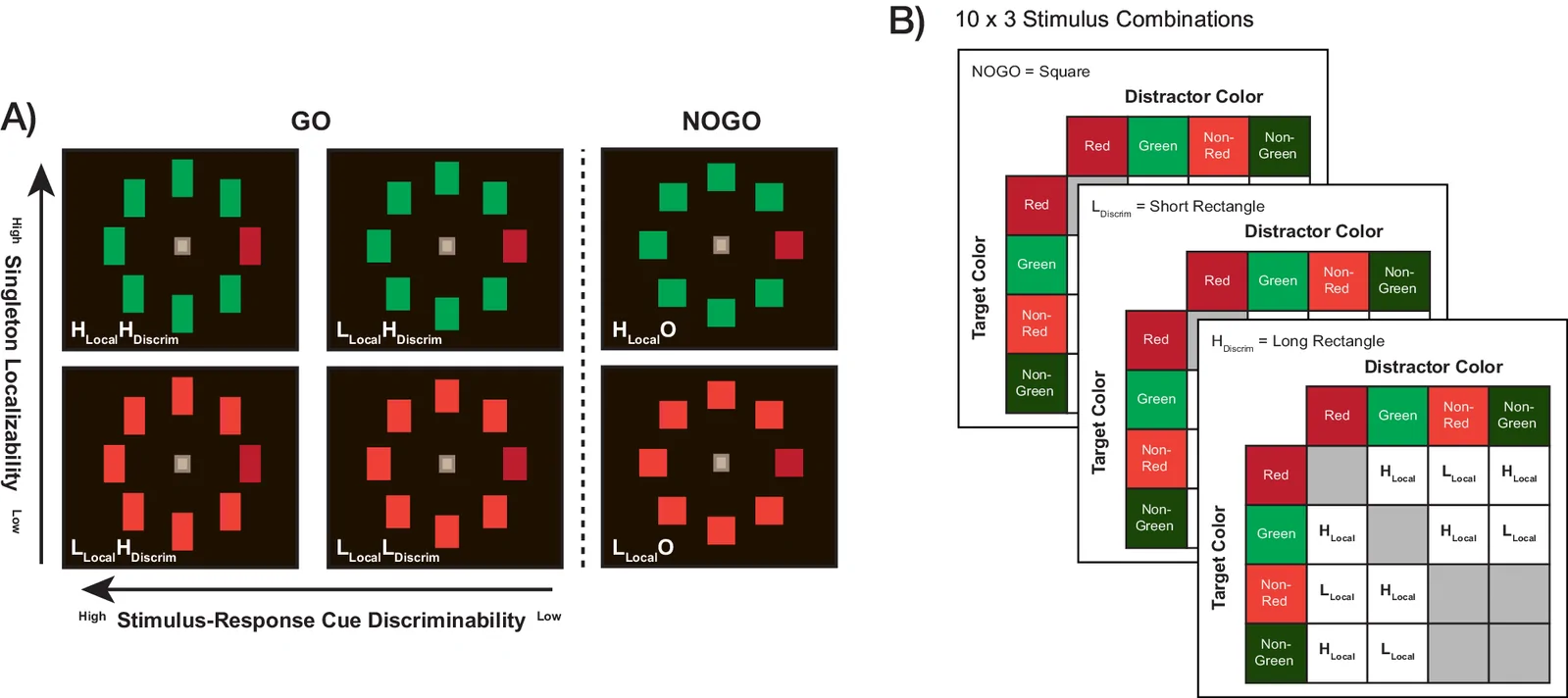
(**A**) Visual search with separately modified singleton localization and stimulus–response cue discrimination. Six basic trial types with red singleton are illustrated. Stimulus shape (elongation) cued the stimulus–response rule: square, NoGo; elongated, Go (horizontal arrow). (**B**) All stimulus combinations.

The efficiency to localize the singleton among distractors and the efficiency to discriminate the stimulus–response cue were separately modified by manipulating visual search stimuli along two dimensions (Figure 1A). Variation of singleton–distractor color similarity manipulated the efficiency to localize the singleton. To discourage a monkey from establishing a target template, singleton and distractor colors were drawn randomly from four chomaticities: red (CIE x = 628, y = 338, Y = 4.4 or x = 604, y = 339, Y = 5.2), off-red (x = 552, y = 399, Y = 4.5 or x = 520, y = 405, Y = 6.6), green (x = 280, y = 610, Y = 4.6 or x = 292, y = 575, Y = 6.1), and off-green (x = 322, y = 558, Y = 4.6 or x = 364, y = 426, Y = 6.8) presented on a dark-gray background (x = 275, y = 228, Y = 0.54 or x = 334, y = 375, Y = 0.6) (Figure 1B). Variation of stimulus elongation manipulated the efficiency to discriminate the stimulus–response cue. Stimuli were randomly drawn from four aspect ratios: 1.0 for NoGo and either 1.2 to 1.4 (less elongated and more difficult) or 2.0 (more elongated and easier) for Go. The area of the stimuli was equated to maintain equal luminance. Orientation was counterbalanced with vertical cuing Go for monkey Da and horizontal cuing Go for Le.

The 2 manipulations × 2 levels of difficulty resulted in four types of Go trials. The easiest was high-efficiency singleton localizability with high-efficiency stimulus–response cue discriminability (H_Local_H_Discrim_). The most difficult was low singleton localizability with low stimulus–response cue discriminability (L_Local_L_Discrim_). Of intermediate difficulty were low singleton localizability with high stimulus–response cue discriminability (L_Local_H_Discrim_) and high singleton localizability with low stimulus–response cue discriminability (H_Local_L_Discrim_). The two NoGo trial types were high-efficiency singleton localizability (H_Local_O) and low-efficiency singleton localizability (L_Local_O). In total, there were 10 singleton–distractor color combinations and three response cues: square, less elongated, and more elongated (Figure 1B; Table 1). The monkeys made three types of errors: localization error, response selection error, and omission error. A localization error occurred when the monkeys shifted their gaze to the distractor instead of the singleton on a Go trial; these trials were flagged immediately upon the gaze landing on any distractors. A response selection error occurred when a monkey failed to maintain fixation at the central stimulus for the required duration on a NoGo trial; these trials were flagged immediately upon the gaze leaving the central stimulus. Omission error trials, in which the monkeys made no saccade on a Go trial, rarely occurred (<0.1% trials for both monkeys) and are not reported here.

**Table 1.** All possible display combinations with the corresponding difficulty level. High difficulty corresponds to low efficiency, and vice versa.

Color			Difficulty	
Singleton	Distractor	Shape	Singleton localizability	Stimulus–response cue discriminability
Red	Off-red	Square	High	NoGo
		Short rectangle	High	High
		Long rectangle	High	Low
	Green	Square	Low	NoGo
		Short rectangle	Low	High
		Long rectangle	Low	Low
	Off-green	Square	Low	NoGo
		Short rectangle	Low	High
		Long rectangle	Low	Low
Off-red	Red	Square	High	NoGo
		Short rectangle	High	High
		Long rectangle	High	Low
	Green	Square	Low	NoGo
		Short rectangle	Low	High
		Long rectangle	Low	Low
Green	Red	Square	Low	NoGo
		Short rectangle	Low	High
		Long rectangle	Low	Low
	Off-red	Square	Low	NoGo
		Short rectangle	Low	High
		Long rectangle	Low	Low
	Off-green	Square	High	NoGo
		Short rectangle	High	High
		Long rectangle	High	Low
Off-green	Red	Square	Low	NoGo
		Short rectangle	Low	High
		Long rectangle	Low	Low
	Green	Square	High	NoGo
		Short rectangle	High	High
		Long rectangle	High	Low

### Offline calibration of eye position signal

We collected online-calibrated eye position signals from the video-oculography system from both monkeys. The eye position signals of Le were also offline calibrated with a second-order polynomial calibration using 16 known fixation locations—eight directions at each of the two eccentricities (6° and 8°)—arranged in a circular array. To account for nonlinearities and cross-axis interactions in the eye position signals, we related the known horizontal and vertical fixation positions, *H* and *V*, to the recorded signals *h* and *v* using the following second-order polynomial model:

$$\begin{array}{c} H=A_{h}+B_{h}h+C_{h}h^{2}+D_{h}hv+E_{h}v \end{array}$$

$$\begin{array}{c} V=A_{v}+B_{v}v+C_{v}v^{2}+D_{v}hv+E_{h}h \end{array}$$

where coefficient *A* represents a baseline offset, *B* represents a linear gain, *C* represents second-order non-linearities, and *D* and *E* account for cross-axis interactions. Calibration parameters were estimated independently for each session using linear least-squares regression. For each fixation location, the endpoint of all correct saccades to that location was averaged to obtain representative (*h*, *v*) values, which were paired with the known fixation positions (*H*, *V*). The fitted calibration parameters were then applied to all eye position data to obtain calibrated eye positions.

### Saccade detection

Saccades were detected using a velocity-based algorithm method developed by Engbert and Kliegl (2003). Eye velocity for horizontal and vertical directions was derived from the raw eye position signals (1-kHz sampling) using the low-pass differentiation algorithm (_1_*f*^3^) described by Usui and Amidror (1982):

$$\begin{array}{c} {\vec{v}}_{n}=\frac{{\vec{x}}_{n+2}+{\vec{x}}_{n+1}-{\vec{x}}_{n-1}-{\vec{x}}_{n-2}}{6\Delta t} \end{array}$$

where ∆*t* is the interval between samples (1 ms). This filter computes the two-point difference between two equally weighted three-sample moving averages, centered on the samples immediately preceding and succeeding the center sample, divided by the 2-ms interval separating them. As a symmetrical filter, it introduces no time delay or phase distortion within the passband (Usui & Amidror, 1982). A multiple λ of the standard deviation of the velocity distribution in each axis was used as the detection threshold. This resulted in a two-dimensional (2D) threshold in the form of an ellipse. We used λ = 3 for both monkeys and all trials, except for λ = 1 to ensure detection of the smaller saccades produced by monkey Da on NoGo error trials. Importantly, the detection threshold was chosen relative to the noise level in each trial. A saccade was defined when the gaze shift velocity exceeded the threshold for a minimum duration τ (12 ms; both monkeys). We implemented this algorithm in MATLAB (MathWorks, Natick, MA), adapting an existing Python implementation (Schwetlick, Schmitthäuser, & Engbert, 2025). For subsequent vigor calculation, the horizontal and vertical velocities were combined into unsigned 2D speed, and peak velocity was taken as the maximum value during the saccade interval.

Saccade initiation was defined as the earliest time at which a qualifying eye movement exceeded the velocity threshold. RT was the time elapsed between array presentation and saccade initiation. Saccade termination was defined as the final time before velocity returned below threshold. Only the first saccade on each trial was considered. Saccade amplitude was calculated as the maximum excursion of eye position between initiation and termination along each axis. This extrema-based measure avoids any postsaccadic oscillations and small corrective movements. In fact, the beginning of the postsaccade oscillation is a criterion to determine saccade offset (Schweitzer & Rolfs, 2022). Maximum excursion was used instead of net displacement (the distance between eye position at initiation and termination, which may capture any corrective adjustment before velocity returned below threshold) because our goal was to quantify the amplitude and vigor of the primary ballistic movement rather than the residual displacement after subsequent corrective adjustments. To be included in analyses, eye movements had to meet the following criteria: (1) saccade initiation within 1000 ms of array presentation, (2) peak velocity lower than 1200°/s, and (3) amplitude between 2° and 12°.

### Saccade vigor calculation

Saccade vigor was quantified by the relationship between saccade amplitude and peak velocity of all task-relevant saccades (Bahill et al., 1975). To compute saccade vigor, we fitted the exponential equation:

$$\begin{array}{c} Peak\;Velocity=V_{0}*\left(1-e^{-Amplitude/C}\right) \end{array}$$

where *V*_0_ is the asymptotic velocity and *C* is the rate constant (Baloh, Sills, Kumley, & Honrubia, 1975; Leigh & Zee, 2015) for the saccade main sequence comprised of all saccades produced in the task for each monkey across all sessions. Because saccades in different directions are produced by different sets of muscles and have different main sequences (Collewijn, Erkelens, & Steinman, 1988a; Collewijn, Erkelens, & Steinman, 1988b) and because of the variation in sensitivity of the video-based eye tracker across saccade direction, we separately fitted exponential function to saccades in each direction. We have also tested an alternative form of the exponential equation with an additional horizontal shift parameter, A_0_ (Gibaldi & Sabatini, 2021; Optican & Robinson, 1980):

$$\begin{array}{c} Peak\;Velocity=V_{0}*\left(1-e^{Amplitude-A_{0}/C}\right) \end{array}$$

The main findings reported below are unchanged when using vigor estimates from the three-parameter functions.

The left panels of Figure 2 show the conventional main sequence for all rightward saccades produced by monkeys Da (Figure 2A) and Le (Figure 2B), with peak velocity plotted as a function of amplitude. Conventionally, the vigor of each saccade is computed as the ratio between the measured velocity and the expected velocity according to the exponential fit based on the amplitude (Reppert et al., 2015); ratios > 1.0 measure saccades with greater than expected vigor, and vice versa.

**Figure 2.**
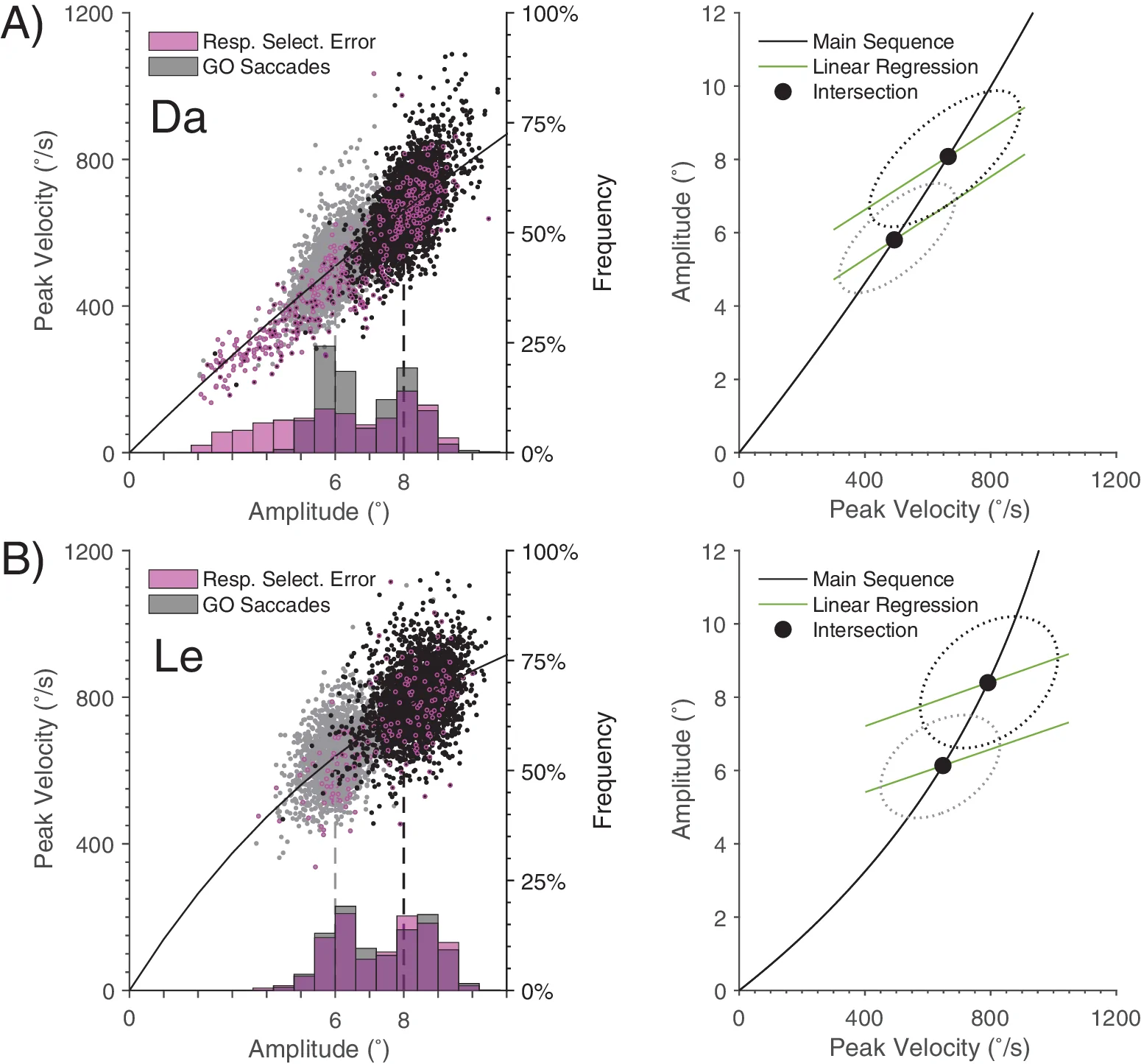
Saccade main sequence for monkey Da (**A**) and Le (**B**). (Left column) Main sequence plots showing all rightward saccades produced during the task across all sessions, with the corresponding exponential fit. Saccades were made to a singleton target at either 6° (light gray) or 8° (black) eccentricity. Saccades from response selection error trials are highlighted with magenta outlines. The left ordinate indicates peak saccade velocity. Overlaid histograms (right ordinate) show the frequency distribution of saccades amplitudes, plotted separately for response selection error on NoGo trials (magenta) and for all Go trials, including both correct and error responses (dark gray). (Right column) Illustration of the method described by Quaia et al. (2000). The inverse of the exponential fitting function is plotted together with linear regression lines for saccades at each eccentricity. The intersection defines the expected peak velocity for saccades of a given direction and eccentricity. Saccade distribution at each eccentricity is represented by the 95th-percentile confidence ellipses.

Classical models of saccade production (e.g., Robinson, 1975) presume that the saccade innervation signal is adjusted online against an internal estimate of the motor error through a feedback loop. Such models predict that, for a given saccade amplitude, any incidental variation in velocity will be compensated during the trajectory of the saccade. In other words, the eye should land on the target regardless of how fast it traveled. Under perfect compensation, amplitude and peak velocity would be uncorrelated among saccades directed to the same target. In fact, though, researchers have found that the amplitude of a series of saccades directed to the same target is positively correlated with their peak speed, and that this additional source of variation cannot be accounted for by the main sequence alone (Quaia et al., 2000). Others have described this variability in saccade amplitude to the same target amplitude as pulse-height noise (Smeets & Hooge, 2003). As a result, a faster saccade to a given target tends to land farther than the intended location. Evaluating the main sequence at this inflated amplitude returns a higher expected velocity, biasing the observed/expected ratio toward 1.0. We addressed this additional source of variation.

Following Quaia et al. (2000), we present the same data with the axes exchanged, plotting amplitude as a function of peak velocity to illustrate the dependence of saccade amplitude on their peak velocity to the same target (Figure 2, right panel). Here, peak velocity is the independent variable because saccade velocity to the same target varies, which affects the landing position. The data were fitted by the inverse of the exponential main sequence function:

$$\begin{array}{c} Amplitude=-\;C*\mathrm{In}\left(1-\frac{Peak\;Velocity}{V_{0}}\right) \end{array}$$

Note that the left and right panels are transpositions of each other describing the same relationship. The latter orientation allowed us to examine whether trial-by-trial variation in movement velocity is compensated by the oculomotor system. Specifically, for each combination of target direction and eccentricity, we fitted a linear regression:

$$\begin{array}{c} Amplitude=m*\;Peak\;Velocity+b \end{array}$$

to the saccades directed toward that location. If variations in saccade velocity were fully compensated (i.e., if the eye landed accurately on target regardless of the movement speed), then the slope of this regression would be zero. For both monkeys, the slope was positive: faster saccades to the same target also traveled farther. Given that amplitude and peak velocity covary among saccades to the same target, the main sequence alone cannot account for all of the variation in the amplitude–peak velocity relationship. Following Quaia et al. (2000), we took the peak velocity at the intersection of the two fits as the expected velocity for a saccade directed toward a target at that specific direction and eccentricity to account for the within-target variation. The vigor of each saccade was then computed as the ratio of its observed peak velocity to this corrected expected velocity.

### Pupil diameter

Video-based eye-tracking systems estimate gaze position by tracking the center of pupil and the corneal reflection. However, during rapid saccadic eye movements, motion of the lens and iris lags behind the motion of the sclera and cornea (Porterfield, 1737; Wade & Tatler, 2005). The sluggish pupil motion relative to ocular rotation results in gaze location estimated from pupil position showing a delayed movement onset, elevated peak velocity, and earlier postsaccadic oscillation (Hooge et al., 2016). Moreover, measurements of saccade kinematics, including amplitude and peak velocity, vary with eye-tracking systems (Hooge, Holmqvist, & Nyström, 2016; Kimmel, Mammo, & Newsome, 2012; Nyström et al., 2016). To determine whether observed variations in saccade velocity can be confounded by systematic variation of pupil size, we assessed whether pupil diameter covaried with saccade vigor and modulated the effects of singleton localizability and stimulus–response cue discriminability. Pupil diameter data was filtered with a third-order Butterworth low-pass filter with a cut-off frequency of 100 Hz. The measurement was in arbitrary units (a.u.) measured at the time of saccade initiation.

### Statistical analyses

We used a chi-square test to assess whether localization error rate and response selection error rate differ significantly across task conditions defined by localizability and cue discriminability. A least significant difference test was used to compare the mean RTs between correct and error trials for each condition. A *t*-test was used to compare the mean vigor between overall correct and localization error saccades, between correct and response selection error saccades, and between localization error and response selection error saccades. Two-way analysis of variance (ANOVA) tests were used to assess the effects of manipulating localizability and discriminability on RTs, saccade vigor, and pupil diameter. Full-factorial generalized linear models (GLMs) were used to assess the relationship of pupil diameter with saccade vigor and the two task factors.

## Results

These results are based on performance measured in 101 sessions (Da: 62 sessions, mean = 2343 trials per session; Le: 39 sessions, mean = 2263 trials per session); 1.53% of trials were removed due to artifacts preventing reliable saccade detection. Analyses on pupil diameter and the associated statistical tests are based on 89 out of 101 sessions (Da, 56 sessions; Le, 33 sessions) where pupil diameter was recorded.

### Correct response times

The correct RTs of both monkeys were separately modified with mutual invariance. The correct RTs of both monkeys varied differentially as a function of the singleton localizability and stimulus–response cue discriminability manipulations (Figure 3, Table 2). RTs were significantly longer on trials with lower singleton localizability (more similar singleton and distractor color) and lower stimulus–response cue discriminability (shorter and more ambiguous shape). Neither monkey showed a significant interaction between singleton localizability and stimulus–response cue discriminability. Notably, the RT difference between the L_Local_ and H_Local_ conditions was smaller in monkey Le than in monkey Da. The variation of RTs across conditions observed here replicated variations reported in 60 sessions (Da, 30; Le, 30) from previous work (Lowe et al., 2019). Unlike the present data, the previous work reported a significant interaction effect of localizability and discriminability on variation in RTs, especially for monkey Da.

**Figure 3.**
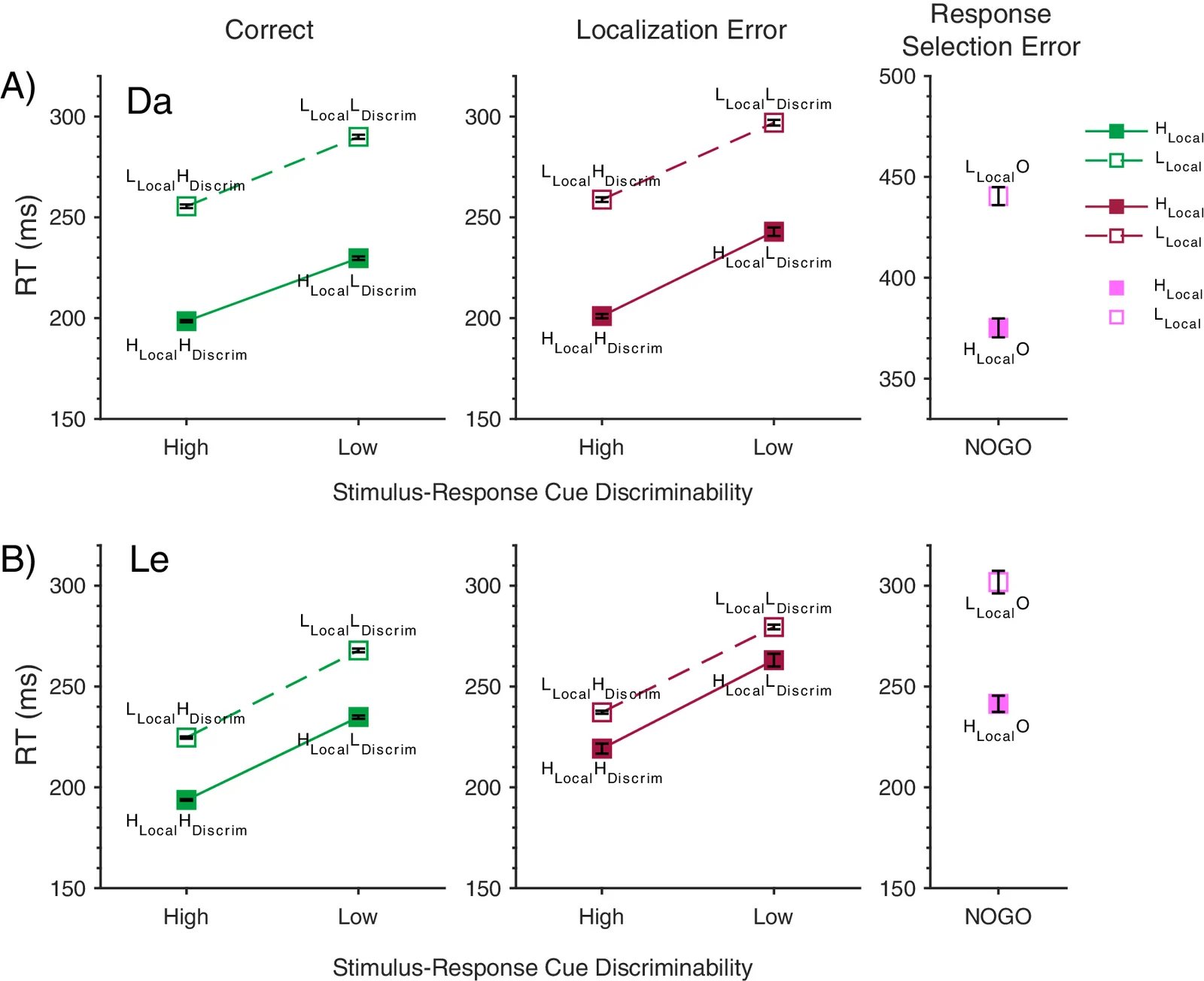
Mean ± SEM RTs for correct (green), localization error on Go trials (red), and response selection responses errors on NoGo trials (magenta) for Da (**A**) and Le (**B**). Correct and localization error trials are shown for the most efficient high singleton localizability and high stimulus–response cue discriminability condition (H_Local_H_Discrim_), the least efficient low singleton localizability and low stimulus–response cue discriminability condition (L_Local_L_Discrim_), and the intermediate conditions (L_Local_H_Discrim_, H_Local_L_Discrim_). Response selection errors with high (H_Local_O) and low (L_Local_O) singleton localizability are plotted in magenta.

**Table 2.** Response time mean ± SEM (ms) and associated ANOVA table. *P*-values ≤ 0.05 are shown in bold. Trial outcomes are italicized.

		Discriminability							
		High	Low	Factor	Sum of squares	*df*	Mean square	*F*	*p*
Monkey Da									
*Correct*									
				Discriminability	0.98 × 10^7^	1	0.98 × 10^7^	1.27 × 10^3^	**<0.001**
				Localizability	3.12 × 10^7^	1	3.12 × 10^7^	4.03 × 10^3^	**<0.001**
Localizability	High	198 ± 0.6	230 ± 0.9	Discriminability × localizability	2.32 × 10^4^	1	2.32 × 10^4^	3.00	0.084
	Low	255 ± 0.9	290 ± 1.0	Error	2.91 × 10^8^	37544	7.74 × 10^3^		
*Localization error*									
				Discriminability	5.40 × 10^6^	1	5.40 × 10^6^	439.35	**<0.001**
				Localizability	1.06 × 10^7^	1	1.06 × 10^7^	858.99	**<0.001**
Localizability	High	201 ± 1.1	243 ± 2.1	Discriminability × localizability	1.16 × 10^4^	1	1.16 × 10^4^	0.95	0.33
	Low	259 ± 1.2	297 ± 1.4	Error	2.62 × 10^8^	21323	1.23 × 10^4^		
*Response selection error*									
				Localizability	4.07 × 10^6^	1	4.07 × 10^6^	102.09	**<0.001**
Localizability	High	375 ± 4.7		Error	1.52 × 10^8^	3812	3.99 × 10^4^		
	Low	440 ± 4.5							
Monkey Le									
*Correct*									
				Discriminability	1.18 × 10^7^	1	1.18 × 10^7^	3.62 × 10^3^	**<0.001**
				Localizability	6.78 × 10^6^	1	6.78 × 10^6^	2.08 × 10^3^	**<0.001**
Localizability	High	194 ± 0.4	235 ± 0.8	Discriminability × localizability	7.10 × 10^3^	1	7.10 × 10^3^	2.18	0.14
	Low	225 ± 0.6	268 ± 0.9	Error	8.67 × 10^7^	26639	3.26 × 10^3^		
*Localization error*									
				Discriminability	2.51 × 10^6^	1	2.51 × 10^6^	7474.24	**<0.001**
				Localizability	4.00 × 10^5^	1	4.00 × 10^5^	75.65	**<0.001**
Localizability	High	219 ± 2.4	263 ± 3.2	Discriminability × localizability	862.20	1	862.20	0.16	0.69
	Low	237 ± 0.8	280 ± 1.2	Error	6.83 × 10^7^	12917	5.29 × 10^3^		
*Response selection error*									
				Singleton localizability	9.83 × 10^5^	1	9.83 × 10^5^	78.94	**<0.001**
Singleton localizability	High	241 ± 4.0		Error	1.44 × 10^7^	1156	1.24 × 10^4^		
	Low	302 ± 5.6							

### Error rates

Overall error rate was 28.3% (Da, 29.7%; Le, 26.1%). The monkeys produced two errors most commonly, and their incidence varied across the 2 × 2 factors (Table 3). Both monkeys made localization errors; that is, the saccade was made to a distractor instead of to the singleton (Da, 36.1% of Go trials; Le, 31.8%). Predictably, these errors were more frequent when singleton localizability was low (L_Local_H_Discrim_ and L_Local_L_Discrim_) (Table 3). A chi-square test showed a significant main effect of singleton localizability on localization error rate. The incidence of localization errors was also significantly higher when stimulus–response cue discriminability was more efficient (H_Local_H_Discrim_ and L_Local_H_Discrim_). For monkey Da, for both levels of singleton localizability, localization error rates were more frequent when stimulus–response cue discriminability was high. For monkey Le, the localization error rate was influenced by stimulus–response cue discriminability only when singleton localizability was low; high stimulus–response cue discriminability produced more localization errors. Response selection errors were failures to maintain fixation on NoGo trials. They were more common for Da (15.6% of NoGo trials) than for Le (9.0%). Both monkeys made more response selection errors when singleton localizability was high (Table 3).

**Table 3.** Error rates for localization error and response selection error. *P*-values ≤ 0.05 are shown in bold. Trial outcomes are italicized.

		Discriminability						
		High	Low	Comparison	χ^2^	*df*, *n*	*p*	Φ
Monkey Da								
*Localization error*								
				H_Local_ vs. L_Local_	2722.71	1, 58,913	**<0.001**	0.215
				H_Discrim_ vs. L_Discrim_	77.96	1, 58,913	**<0.001**	0.036
Localizability	High	23.6%	19.7%	H_Local_H_Discrim_ vs. H_Local_L_Discrim_	42.72	1, 19,754	**<0.001**	0.047
	Low	45.2%	41.9%	L_Local_H_Discrim_ vs. L_Local_L_Discrim_	42.12	1, 39,159	**<0.001**	0.033
*Response selection error*								
Localizability	High	18.1%		H_Local_ vs. L_Local_	89.35	1, 26,909	**<0.001**	0.058
	Low	13.9%						
Monkey Le								
*Localization error*								
				H_Local_ vs. L_Local_	6604.52	1, 42,154	**<0.001**	0.396
				H_Discrim_ vs. L_Discrim_	27.56	1, 42,154	**<0.001**	0.026
Localizability	High	9.6%	9.3%	H_Local_H_Discrim_ vs. H_Local_L_Discrim_	0.46	1, 17,036	0.50	0.005
	Low	48.6%	45.3%	L_Local_H_Discrim_ vs. L_Local_L_Discrim_	26.50	1, 25,118	**<0.001**	0.0032
*Response selection error*								
Localizability	High	11.1%		H_Local_ vs. L_Local_	78.69	1, 14,356	**<0.001**	0.074
	Low	6.8%						

### RTs on errors trials

RTs varied between correct and error trials. The RTs of localization errors were consistently longer than those of correct RTs. For monkey Da, relative to correct RTs, localization error RTs were significantly longer in the H_Local_L_Discrim_ (mean difference = 13 ms; 95% confidence interval [CI], 8–18; *p* < 0.001), L_Local_H_Discrim_ (mean difference = 3 ms; 95% CI, 1–6; *p* = 0.015), and L_Local_L_Discrim_ (mean difference = 7 ms; 95% CI, 4–10; *p* < 0.001) conditions. Localization error RTs were also longer in the H_Local_H_Discrim_ condition, but the difference was not significant (*p* = 0.27). For Le, localization error RTs were significantly longer than correct RTs in all conditions: H_Local_H_Discrim_ (mean difference = 25 ms; 95% CI, 21–30; *p* < 0.001), H_Local_L_Discrim_ (mean difference = 28 ms; 95% CI, 24–32; *p* < 0.001), L_Local_H_Discrim_ (mean difference = 13 ms; 95% CI, 10–15; *p* < 0.001), and L_Local_L_Discrim_ (mean difference = 12 ms; 95% CI, 9–14; *p* < 0.001). The magnitude of the difference was relatively small in Da, ranging from 3 ms to 13 ms. It was larger in Le, ranging from 12 ms to 28ms.

The pattern of RTs of response selection errors when gaze was shifted on NoGo trials differed between the two monkeys. For monkey Da, mean RTs on response selection error trials were nearly twice of those observed on correct trials or on localization error trials, under both high and low singleton localizability. For monkey Le, mean RTs on response selection error trials were more comparable in scale to the correct and localization error trials. Consistent with the patterns observed for correct and localization error trials, the RT difference between the L_Local_ and H_Local_ conditions was again smaller in monkey Le than in monkey Da.

For both monkeys, the variation of RT of localization errors across the singleton localizability and stimulus–response cue discriminability levels mirrored that observed on correct trials. Separate modifiability by both the singleton localizability and stimulus–response cue discriminability manipulations was observed for the RTs of saccades directed to a distractor. No significant interaction between the two factors was observed. The RTs of response selection errors varied significantly with singleton localizability, with low singleton localizability showing higher RTs than high singleton localizability (Figure 3; Table 2). Overall, the effects of singleton localizability and stimulus–response cue discriminability were seen across all trial types.

### Saccade vigor

Figure 2 shows the main sequence of saccade velocity as a function of amplitude with the exponential regression for all rightward saccades from monkey Da and Le. The distribution of saccade amplitudes is also presented in a histogram, illustrating the two groups of saccades produced to search arrays with eccentricities of 6° and 8°. The saccade amplitude distribution of monkey Da was left skewed, because of the hypometric saccades produced in NoGo response selection error trials. In contrast, monkey Le rarely made hypometric saccades.

To examine the relationship between vigor and RT, all saccades were vincentized into 10 bins, and the mean RTs and mean vigor (± *SEM*) were plotted for each quantile (Figure 4). Mean saccade vigor of all saccades, irrespective of latency, was consistently ordered for both monkeys: highest for correct trials (mean for Da, 1.03; Le, 1.01), intermediate for localization error trials (Da, 0.98; Le, 0.98) and lowest response selection error trials (Da, 0.80; Le, 0.92). Concomitantly, mean RTs, irrespective of trial types, were shortest for correct trials (weighted mean RT for Da, 248 ms; Le, 229 ms), longer for localization errors (Da, 265 ms; Le, 255 ms), and longest for response selection errors (Da, 408 ms; Le, 264 ms). Thus, there was an inverse relationship between vigor and RTs across both monkeys for correct and localization error saccades. For monkey Da but not Le, saccade vigor decreased with latency of response selection errors. A linear model predicting vigor with trial type and RT as factors revealed a significant main effect of both trial type, Da, *F*(2, 62,683) = 3008.00, *p* < 0.001; Le, *F*(2, 40,716) = 342.93, *p* < 0.001, and RTs, Da, *F*(1, 62,683) = 3489.60, *p* < 0.001; Le, *F*(1, 40,716) = 44.80, *p* < 0.001, with a significant interaction between RTs and vigor across trial types, Da, *F*(2, 62,683) = 369.01, *p* < 0.001; Le, *F*(2, 40,716) = 83.83, *p* < 0.001. For monkey Da, vigor and RTs were inversely related primarily when localizability was varied; they mostly co-varied with changes in discriminability. The reverse relationships were generally observed in monkey Le.

**Figure 4.**
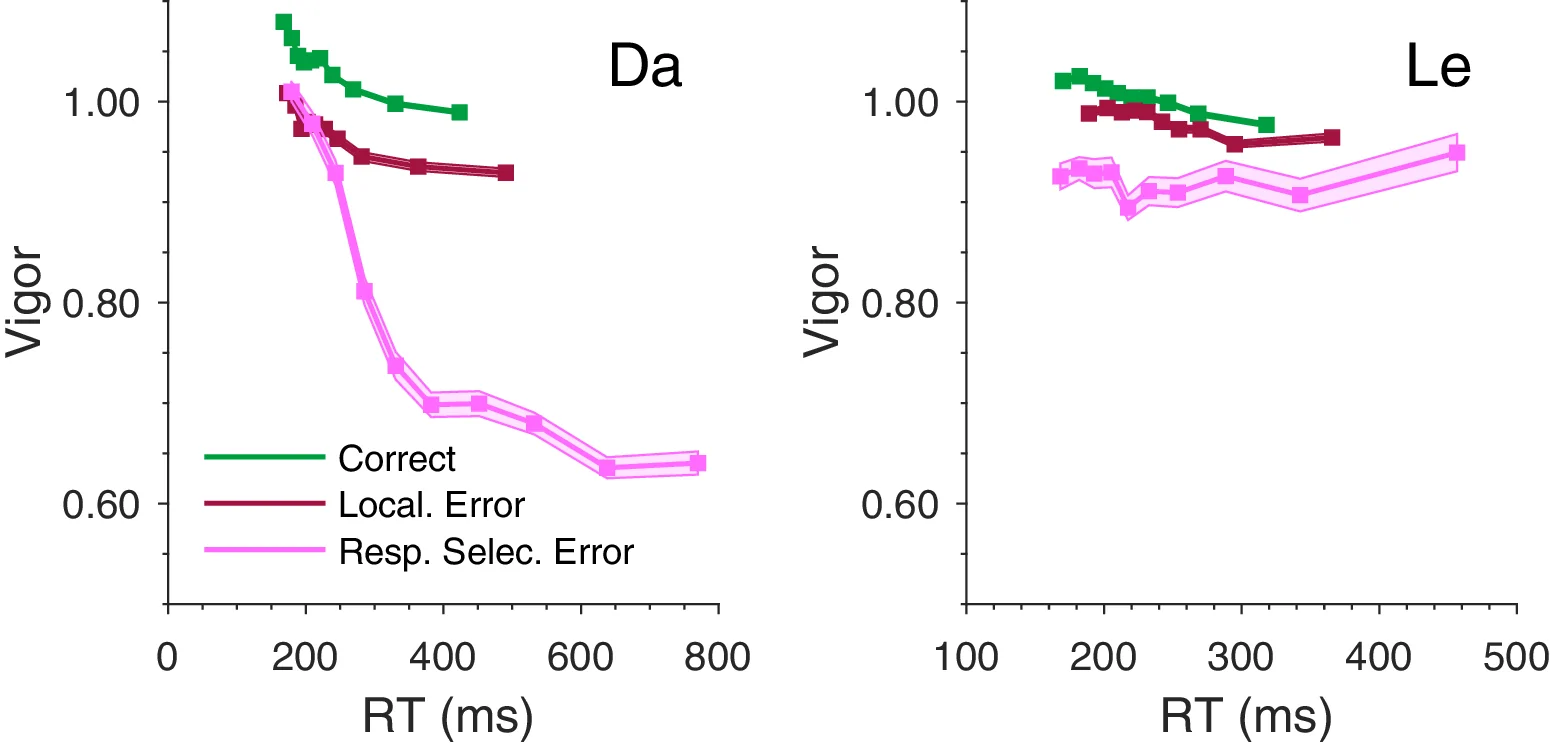
Mean ± SEM of saccade vigor as a function of vincentized RT for Da (left) and Le (right).

### Separate modifiability of saccade vigor

Unlike RT, saccade vigor was not separately modified with mutual invariance. A two-way ANOVA with singleton localizability and stimulus–response cue discriminability was used to assess saccade vigor across the 2 × 2 task conditions, separately for correct and error trials. Singleton localizability and stimulus–response cue discriminability affected saccade vigor differently for the two monkeys (Figure 5; Table 4). For monkey Da, vigor was greatest when singleton localizability was most efficient (H_Local_), regardless of stimulus–response cue discriminability (Figure 5A). On both correct and localization error trials, vigor varied significantly with both singleton localizability and stimulus–response cue discriminability. The singleton localizability effect had the largest effect size. On correct trials, there was a significant interaction between these factors; the difference in vigor between high and low singleton localizability was smaller with low stimulus–response cue discriminability relative to high. On response selection error trials, the singleton localizability effect remained significant.

**Figure 5.**
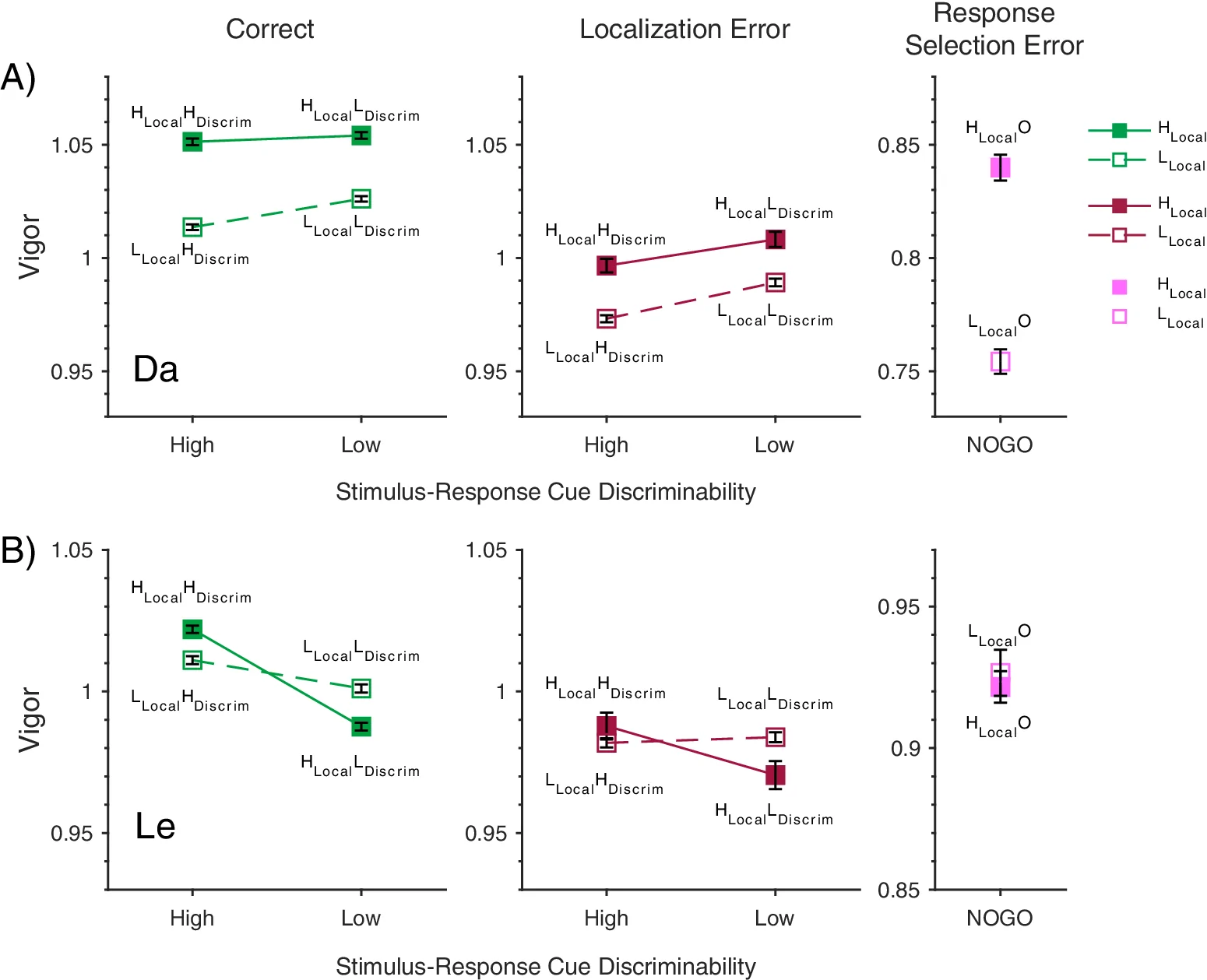
Mean ± SEM vigor for correct (green), localization error on Go trials (red), and response selection error in NoGo trials (magenta) for Da (**A**) and Le (**B**). Correct and localization error trials are shown for the most efficient high singleton localizability and high stimulus–response cue discriminability condition (H_Local_H_Discrim_), the least efficient low singleton localizability and low stimulus–response cue discriminability condition (L_Local_L_Discrim_), and the intermediate conditions (L_Local_H_Discrim_, H_Local_L_Discrim_). Response selection errors with high (H_Local_O) and low (L_Local_O) singleton localizability are plotted in magenta.

**Table 4.** Saccade vigor mean ± SD (ms) and associated ANOVA table. *P*-values ≤ 0.05 are shown in bold. Trial outcomes are italicized.

		Discriminability								
		High	Low	Factor	Sum of squares	*df*	Mean square	*F*	*p*	Partial η^2^
Monkey Da										
*Correct*										
				Discriminability	0.53	1	0.53	32.34	**<0.001**	<0.001
				Localizability	9.79	1	9.79	595.43	**<0.001**	0.016
Localizability	High	1.051 ± 0.002	1.054 ± 0.002	Discriminability × localizability	0.22	1	0.22	13.13	**<0.001**	<0.001
	Low	1.014 ± 0.001	1.026 ± 0.001	Error	617.56	37544	0.02			
*Localization error*										
				Discriminability	0.64	1	0.64	29.07	**<0.001**	0.001
				Localizability	1.52	1	1.52	68.83	**<0.001**	0.003
Localizability	High	0.997 ± 0.003	1.008 ± 0.003	Discriminability × localizability	0.02	1	0.02	0.74	0.389	<0.001
	Low	0.973 ± 0.002	0.989 ± 0.002	Error	471.30	21323	0.02			
*Response selection error*										
Localizability	High	0.840 ± 0.006		Singleton localizability	6.99	1	6.99	118.00	**<0.001**	0.030
	Low	0.754 ± 0.005		Error	225.80	3812	0.06			
Monkey Le										
*Correct*										
				Discriminability	3.26	1	3.25	252.82	**<0.001**	0.009
				Localizability	0.01	1	0.01	0.84	0.360	<0.001
Localizability	High	1.022 ± 0.001	0.988 ± 0.001	Discriminability × localizability	0.98	1	0.98	76.43	**<0.001**	0.003
	Low	1.011 ± 0.001	1.001 ± 0.001	Error	343.25	26639	0.01			
*Localization error*										
				Discriminability	0.08	1	0.08	4.92	**0.027**	<0.001
				Localizability	0.02	1	0.02	1.15	0.284	<0.001
Localizability	High	0.988 ± 0.005	0.971 ± 0.005	Discriminability × localizability	0.13	1	0.13	7.84	**0.005**	<0.001
	Low	0.982 ± 0.002	0.984 ± 0.002	Error	208.44	12,917	0.02			
*Response selection error*										
Localizability	High	0.922 ± 0.006		Localizability	0.01	1	0.01	0.27	0.603	<0.001
	Low	0.927 ± 0.008		Error	28.63	1,156	0.02			

For monkey Le, on both correct and localization error trials, vigor varied significantly with stimulus–response cue discriminability but not singleton localizability (Figure 5B). On correct and localization error trials, the ANOVA showed a significant interaction between stimulus–response cue discriminability and singleton localizability. The interaction between these two factors indicates that vigor was reduced with lower stimulus–response cue discriminability, particularly in trials with high-efficiency singleton localizability. On response error trials, singleton localizability had no significant effect on vigor.

In summary, the two monkeys exhibited distinct vigor modulation patterns. For monkey Da, both singleton localizability and stimulus–response cue discriminability consistently influenced saccade vigor across correct and error trials, consistent with the pattern of variation of RTs across conditions. For monkey Le, stimulus–response cue discriminability was the primary factor influencing saccade vigor on correct and localization error trials. Singleton localizability, which modulated RTs less, influenced vigor only indirectly through an interaction with stimulus–response cue discriminability. On response selection error trials, singleton localizability modulated vigor for monkey Da but had no discernible effect for monkey Le.

### Variation of pupil diameter with singleton localizability and stimulus–response cue discriminability

We first examined whether pupil diameter varied with the two task factors (Figure 6). A two-way ANOVA on pupil diameter at saccade initiation with singleton localizability and stimulus–response cue discriminability as factors showed that pupil diameter varied more with the task factors in monkey Da than in monkey Le (Table 5). In monkey Da, pupil diameter on correct trials increased with singleton localizability and decreased with stimulus–response cue discriminability, with no significant interaction. On localization error trials, both factors remained significant with no interaction; however, pupil diameter decreased with singleton localizability and increased with stimulus–response cue discriminability. On response selection error trials, singleton localizability was not a significant predictor of pupil diameter. In monkey Le, pupil diameter varied significantly with stimulus–response cue discriminability only on correct trials, with no significant effects of either factor on localization error or response selection error trials.

**Figure 6.**
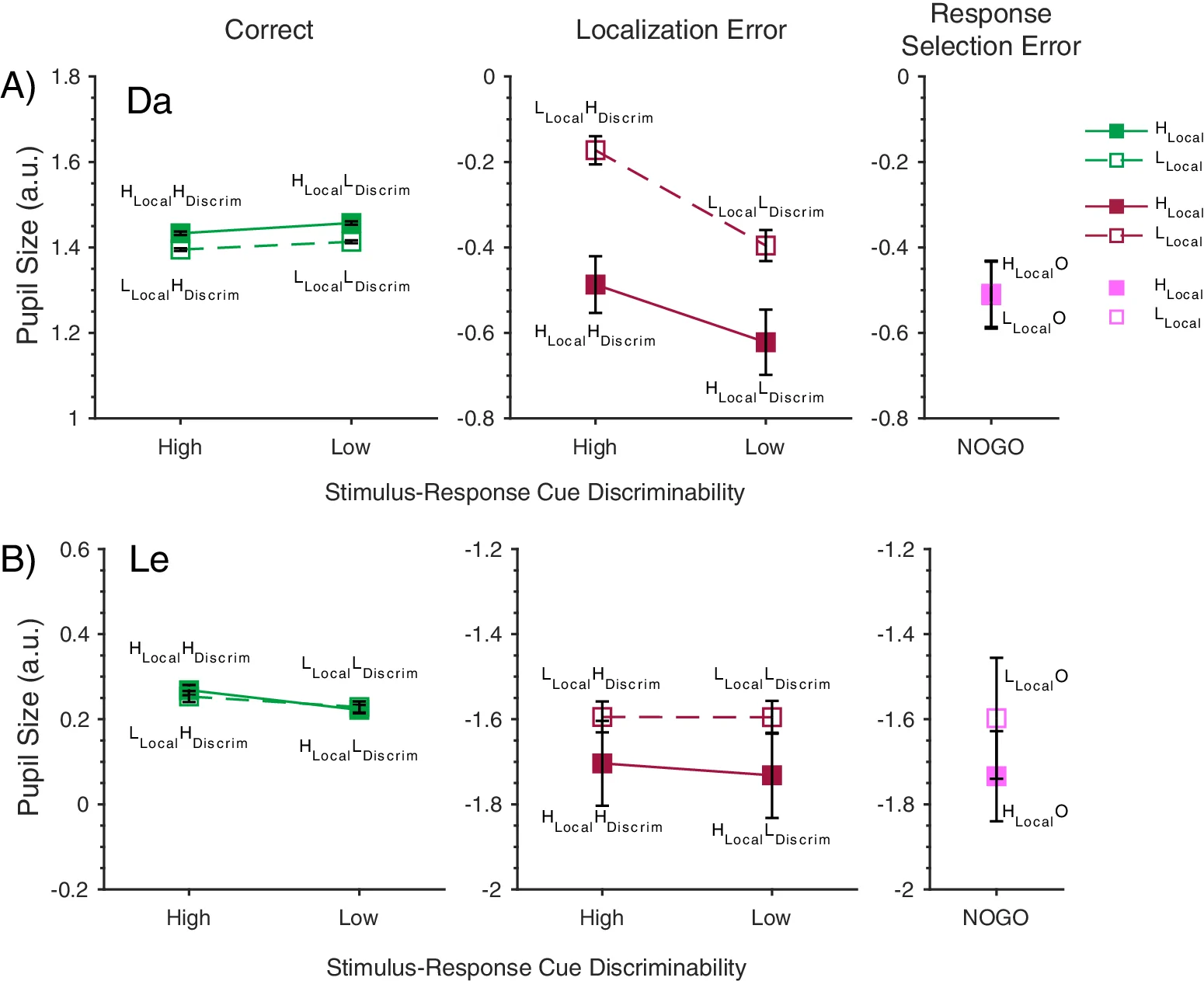
Mean ± SEM pupil size for correct (green), localization error on Go trials (red), and response selection responses errors on NoGo trials (magenta) for Da (**A**) and Le (**B**). Pupil size is baseline corrected. Correct and localization error trials are shown for the most efficient high singleton localizability and high stimulus–response cue discriminability condition (H_Local_H_Discrim_), the least efficient low singleton localizability and low stimulus–response cue discriminability condition (L_Local_L_Discrim_), and the intermediate conditions (L_Local_H_Discrim_, H_Local_L_Discrim_). Response selection errors with high (H_Local_O) and low (L_Local_O) singleton localizability are plotted in magenta.

**Table 5.** Two-way ANOVA on pupil diameter with singleton localizability and stimulus–response cue discriminability as factors. *P*-values ≤ 0.05 are shown in bold. Trial outcomes are italicized.

Factor	Sum of squares	*df*	Mean square	*F*	*p*	Partial η^2^
Monkey Da						
*Correct*						
Discriminability	3.64	1	3.64	32.37	**<0.001**	0.004
Localizability	13.67	1	13.67	121.38	**<0.** **001**	<0.001
Discriminability × localizability	0.06	1	0.06	0.57	0.450	<0.001
Error	3,781.56	33,569	0.11			
*Localization error*						
Discriminability	99.22	1	99.22	10.55	**0.** **001**	0.001
Localizability	226.25	1	226.25	24.06	**<0.001**	<0.001
Discriminability × localizability	6.01	1	6.01	0.64	0.424	<0.001
Error	1.83 × 10^5^	19,455	9.41			
*Response selection error*						
Localizability	0.01	1	0.01	8.64 × 10^−4^	0.977	<0.001
Error	3.52 × 10^4^	3,403	10.34			
Monkey Le						
*Correct*						
Discriminability	7.06	1	7.06	8.26	**0.** **004**	<0.001
Localizability	0.09	1	0.09	0.11	0.740	<0.001
Discriminability × localizability	0.68	1	0.68	0.79	0.374	<0.001
Error	1.92 × 10^4^	22,444	0.86			
*Localization error*						
Discriminability	0.25	1	0.25	0.04	0.845	<0.001
Localizability	16.86	1	16.86	2.54	0.111	<0.001
Discriminability × localizability	0.22	1	0.22	0.03	0.855	<0.001
Error	7.18 × 10^4^	10,802	6.65			
*Response selection error*						
Localizability	4.29	1	4.29	0.58	0.447	
Error	7,584.60	1,024	7.41			

Together, although pupil diameter varied with task difficulty in both monkeys, these effects were inconsistent across trial types and subjects, all with very small effect sizes. Notably, several effects observed in vigor (Figure 5; Table 4) were not mirrored by corresponding effects in pupil diameter (Figure 6; Table 5), including the significant interaction between the two task factors on correct trials in both monkeys and on localization error trials in monkey Le, as well as the significant effect of singleton localizability on response selection error trials in monkey Da. This dissociation suggests that the observed effects of task factors on saccade vigor cannot be fully attributed to pupil-related modulation.

### Effects of task factors on saccade vigor independent of pupil diameter

To assess the effects of singleton localizability and stimulus–response cue discriminability on saccade vigor while accounting for pupil diameter, we used a GLM with the main effects of localizability (two levels), discriminability (two levels), and pupil diameter (continuous), along with all two-way and three-way interactions. To determine the appropriate model specification, we first tested the homogeneity of regression slopes assumption by examining whether pupil diameter interacted significantly with the two task factors.

The assumption was violated on correct trials for both monkeys. On correct trials, pupil diameter interacted significantly with both task factors for monkey Da, with localizability × pupil *F*(1, 33,565) = 5.17, *p* = 0.023, and discriminability × pupil *F*(1, 33,565) = 7.51, *p* = 0.006; for monkey Le, localizability × pupil *F*(1, 22,440) = 7.17, *p* = 0.007, and discriminability × pupil *F*(1, 22,440) = 8.56, *p* = 0.003. This indicates that the relationship between pupil diameter and saccade vigor varied across conditions on correct trials. On error trials, no interactions reached significance for either monkey (all *p* > 0.14). To allow direct comparison across trial types, we retained the full interaction model for both correct and error trials. Full results are presented in Table 6.

**Table 6.** General linear model examining the effects of singleton localizability, stimulus–response cue discriminability, and pupil diameter on saccade vigor. *P*-values ≤ 0.05 are shown in bold. Trial outcomes are italicized.

Factor	Sum of squares	*df*	Mean square	*F*	*p*
Monkey Da					
*Correct*					
Localizability	7.00	1	7.00	458.13	**<0.001**
Discriminability	0.41	1	0.41	27.10	**<0.001**
Pupil diameter	39.54	1	39.54	2.59 × 10^3^	**<0.001**
Localizability × discriminability	0.24	1	0.24	15.72	**<0.001**
Localizability × pupil diameter	0.08	1	0.08	5.17	**0.** **023**
Discriminability × pupil diameter	0.11	1	0.11	7.51	**0.** **006**
Discriminability × localizability × pupil diameter	0.02	1	0.02	1.06	0.303
Error	512.88	33565	0.02	1	0.500
*Localization error*					
Localizability	1.33	1	1.33	60.93	**<0.001**
Discriminability	1.11	1	1.11	50.78	**<0.001**
Pupil diameter	2.30	1	2.30	104.97	**<0.001**
Localizability × discriminability	0.01	1	0.01	0.40	0.528
Localizability × pupil diameter	0.01	1	0.01	0.30	0.585
Discriminability × pupil diameter	0.01	1	0.01	0.40	0.526
Discriminability × localizability × pupil diameter	0.08	1	0.08	3.62	0.057
Error	425.86	19,451	0.02	1	0.500
*Response selection error*					
Localizability	6.48	1	6.48	110.56	**<0.001**
Pupil diameter	0.76	1	0.76	12.95	**<0.001**
Localizability × pupil diameter	0.13	1	0.13	2.17	0.141
Error	199.26	3401	0.06	1	0.500
Monkey Le					
*Correct*					
Localizability	0.03	1	0.03	1.98	0.159
Discriminability	2.82	1	2.82	219.68	**<0.001**
Pupil diameter	2.20	1	2.20	171.39	**<0.001**
Localizability × discriminability	0.82	1	0.82	64.11	**<0.001**
Localizability × pupil diameter	0.09	1	0.09	7.17	**0.** **007**
Discriminability × pupil diameter	0.11	1	0.11	8.56	**0.** **003**
Discriminability × localizability × pupil diameter	5.95 × 10^−4^	1	5.95 × 10^−4^	0.05	0.830
Error	288.14	22,440	0.01	1	0.500
*Localization error*					
Localizability	0.04	1	0.04	2.53	0.112
Discriminability	5.95 × 10^−4^	1	5.95 × 10^−4^	0.04	0.849
Pupil diameter	0.01	1	0.01	0.47	0.492
Localizability × discriminability	0.22	1	0.22	13.16	**<0.001**
Localizability × pupil diameter	0.00	1	0.00	0.08	0.773
Discriminability × pupil diameter	0.02	1	0.02	1.49	0.222
Discriminability × localizability × pupil diameter	0.02	1	0.02	1.11	0.292
Error	177.36	10,798	0.02	1	0.500
*Response selection error*					
Localizability	0.01	1	0.01	0.59	0.442
Pupil diameter	0.01	1	0.01	0.26	0.611
Localizability × pupil diameter	0.00	1	0.00	0.11	0.743
Error	25.45	1,022	0.02	1	0.500

For monkey Da on correct trials, all three predictors showed significant main effects. Among the two-way interactions, the localizability × discriminability interaction, the discriminability × pupil diameter interaction, and the localizability × pupil diameter interaction were significant. The three-way interaction of localizability, discriminability, and pupil diameter was not significant. Thus, vigor varied independently and jointly with singleton localizability and stimulus–response cue discriminability beyond any pupil diameter-mediated effects on correct trials.

On localization error trials, the saccade vigor of monkey Da varied independently with singleton localizability, stimulus–response cue discriminability, and pupil diameter. No interactions reached significance. On response selection error trials, both singleton localizability and pupil diameter showed significant main effects without a significant interaction. These patterns are consistent with the two-way ANOVA results in Table 4, confirming that the effects of task factors on vigor persist after statistically controlling for pupil diameter.

For monkey Le, the results were partially consistent with those of monkey Da (Table 6). On correct trials, stimulus–response cue discriminability and pupil diameter showed significant main effects, as did the localizability × discriminability interaction, the localizability × pupil diameter interaction, and the discriminability × pupil diameter interaction. The three-way interaction was not significant. Notably, the main effect of singleton localizability was not significant, suggesting that singleton localizability does not independently shift overall vigor levels, but instead modulates vigor through interactions with both stimulus–response cue discriminability and pupil diameter.

On localization error trials, only the localizability × discriminability interaction was significant, and all main effects were non-significant. This contrasts with the two-way ANOVA results in Table 4, where stimulus–response cue discriminability showed a significant main effect, suggesting that controlling for pupil diameter reduced the independent effect of stimulus–response cue discriminability on localization error trials, leaving only the interaction effect. On response selection error trials, neither singleton localizability nor pupil diameter reached significance, and there was no significant interaction.

Taken together, these results reveal two consistent findings across monkeys. First, pupil diameter varied with saccade vigor. Its effect was significant across all three trial types in monkey Da and on correct trials for monkey Le. The relationship between pupil diameter and vigor varied across task conditions on correct trials for both monkeys. Second, controlling for pupil diameter did not eliminate the variation of saccade vigor with the two task factors. In monkey Da, both singleton localizability and stimulus–response cue discriminability exerted significant independent effects on vigor across correct and error trials. In monkey Le, stimulus–response cue discriminability showed a significant main effect on correct trials, whereas singleton localizability influenced saccade vigor primarily through its interaction with discriminability rather than as an independent predictor. A significant singleton localizability × stimulus–response cue discriminability interaction was present in both correct and localization error trials for monkey Le.

In conclusion, although pupil diameter accounted for a portion of the variance in saccade vigor, and its relationship with vigor varied across task conditions and animals, significant effects of singleton localizability and stimulus–response cue discriminability persisted after statistically controlling for pupil diameter.

## Discussion

Stochastic accumulator models of decision-making treat decision and action as independent, serial processes because the models are conceived as describing the process producing a choice response by any effector. In contrast, we found that saccade velocity varied systematically with the factors manipulated the efficiency of two, concurrent decision processes. This finding aligns with growing evidence that manual and ocular response kinematics and dynamics can be influenced by preceding evidence accumulation processes (Dendauw et al., 2024; Reppert, 2025; Seideman et al., 2018; Servant et al., 2021; Wispinski et al., 2020). Our findings are consistent with and add to the previous studies demonstrating that saccade vigor is influenced by reward and motivation (Takikawa et al., 2002) and related variables such as confidence (Seideman et al., 2018), urgency (Thura, 2020), subjective reward (Reppert et al., 2015), effort (Shadmehr et al., 2019), and speed–accuracy trade-off (Reppert et al., 2018b). Stronger signal strength with longer processing time is associated with more vigorous saccades (Seideman et al., 2018) even if they are not relevant for the task (Joo, Katz, & Huk, 2016; Matsumiya & Furukawa, 2023). Generally, saccades are faster when subjects are more motivated and when the expected value or probability of reward is high. Faster movement is thought to increase the rate of reward by reducing temporal costs imposed by movement duration (Choi, Vaswani, & Shadmehr, 2014; Haith et al., 2012; Schütz & Stewart, 2025; Shadmehr, 2010; Shadmehr & Ahmed, 2021).

### Relationship between decision speed and movement vigor

Coupling between decision speed and movement vigor has been cited as evidence for the co-regulation of decision and action processes (Fievez et al., 2024; Reppert, 2025; Thura, Haith, Derosiere, & Duque, 2025). Sometimes, decision and movement speed can also compensate for each other (Saleri & Thura, 2024). The presence or absence of co-regulation is contingent on task incentives (Fievez et al., 2024). Both monkeys exhibited the most vigorous saccades on trials with the fastest RTs, and mean vigor decreased as RTs increased. This inverse relationship was observed on correct and both types of error trials in monkey Da and correct and localization error trials in monkey Le. A prior study of visual search with instructed speed–accuracy trade-off found that saccade vigor varied with RT under the fast but not the accurate instruction (Reppert et al., 2018b; see also Carsten, Fievez, & Duque, 2023). Hence, one interpretation of the present results is that the two monkeys performed this task with an emphasis of speed over accuracy. This interpretation is consistent with their relatively high error rates.

Although we observed an overall co-variation of decision and movement speed in both monkeys, factorial manipulation of the two decision processes affected RT and saccade vigor differently. Manipulating the efficiency of singleton localization and of the stimulus–response cue discrimination process had an overall additive effect on mean RTs for both monkeys, where increases in localizability and stimulus–response cue discriminability each produced faster RTs. However, the pattern was less consistent for vigor and differed between monkeys. After controlling for effects of pupil size, both factors remained significant predictors of vigor for monkey Da across correct and error trials. For monkey Le, stimulus–response cue discriminability had independent effects on vigor, whereas manipulating localizability had no significant effect on vigor by itself. This suggests that not all sources of RT variation are equally coupled to movement vigor. Future studies of co-regulation should investigate the contributions of distinct decision stages.

### Individual variability in performance

As reported previously (Lowe et al., 2019), the two monkeys exhibited idiosyncratic differences in their patterns of performance. Training history may account for this. Whereas this was the only task performed by monkey Le, monkey Da had performed several visual search tasks prior to this study (Cosman, Lowe, Zinke, Woodman, & Schall, 2018; Heitz & Schall, 2012; Reppert et al., 2018b). Such differences offer experimental opportunities with neurophysiological data (e.g., Lowe & Schall, 2019). Distinct patterns of error rates and saccade vigor across monkeys indicated individual differences in decision-making strategies, which can evolve with training (Lowe et al., 2019). Individual variability across monkeys has been observed in other decision-making tasks (e.g., Saleri & Thura, 2024), and previous research has described individual differences in movement vigor generally (Reppert et al., 2018a).

### Models of decision-making and response production

Stochastic accumulator models are the current dominant theoretical framework of perceptual decision-making. They treat motor responses as merely the output of the preceding evidence accumulation process reaching a decision threshold. Canonical forms of these models incorporate no interaction between decision variables and response production parameters. Afferent and efferent stages before and after the evidence accumulation process are reduced to a single non-decisional residual component (Ratcliff, 1978; Ratcliff, 2013; Ratcliff et al., 2016). However, studies suggest that non-decision time parameters are unlikely to be only sensorimotor delays (Bompas, Sumner, & Hedge, 2025; Smith & Lilburn, 2020; Weindel, Gajdos, Burle, & Alario, 2021). More generally, traditional models of decision-making based on discrete RTs and accuracy responses (Forstmann et al., 2016; McClelland, 1979; Ratcliff, 1978; Ratcliff et al., 2016) cannot capture internal processes occurring concurrently and continuously (Cisek, 2006; Gallivan et al., 2018; Song & Nakayama, 2009; see also Schall, 2019).

Other models have been formulated to approach greater neurobiological plausibility. For example, the interaction between neurons representing visual salience and neurons preparing to initiate saccades has been articulated by a gated accumulator model (Purcell et al., 2010; Purcell, Schall, Logan, & Palmeri 2012). This model demonstrates how the neurons preparing saccades correspond to evidence accumulators that trigger saccade initiation (see also Middlebrooks, Zandbelt, Logan, Palmeri, & Schall, 2020; Ratcliff, Hasegawa, Hasegawa, Smith, & Segraves, 2007). This functional attribution is consistent with anatomical observations that these pre-movement evidence accumulation neurons, which are found in structures such as the frontal eye field and superior colliculus, send axons to terminate in brainstem circuits responsible for generating saccades. The understanding of the brainstem saccade circuit is intricate and precise (e.g., Daye, Optican, Roze, Gaymard, & Pouget, 2013; Quaia, Lefèvre, & Optican, 1999). However, we do not know how the reaching of a threshold by pre-movement neurons causes the initiation and production of saccades (Schall & Paré, 2021). The current findings demonstrate that this linkage is leaky and offer data that would usefully constrain a new integrative model that formally links the evidence accumulator with the brainstem saccade circuit.

Other recent studies and models have explored how decision-making processes can interact with the sensorimotor system to produce systematic covariation in RTs and execution (Gallivan et al., 2018; Wispinski et al., 2020). This covariation can be explained by models that incorporate dynamics of both evidence accumulation and responses execution (Dendauw et al., 2024; Molano-Mazón et al., 2024; Servant et al., 2021). If decision and motor output are not strictly sequential processes, then mathematical models must also predict the rate of muscular contraction and explain the kinematics of movement production in relation to decision processes.

Importantly, our results suggest that movement vigor is not simply a read out of the accumulated decision evidence at movement onset, and that distinct decision stages can influence RTs and motor output differently. Therefore, future models of decision-making should incorporate functionally distinct stages with differential influences on the motor system. Predictions of RTs and of movement kinematics will depend on which stages in the decision hierarchy are influenced by a task manipulation.

The present study focused on eye movements, specifically saccade vigor as it relates to RTs. However, it is important to note that a 2 × 2 factorial design provides a powerful framework for dissociating additional covert processing stages underlying different movement types, beyond eye movements. Other aspects of motor execution, such as response duration in keypress tasks, may offer further insight into these latent decision-making stages (Pfister, Neszmélyi, & Kunde, 2023).

Mechanistically, variation in velocity for saccades of a given amplitude entails variation in the pulse innervating the extraocular muscles. This pulse of activity (or burst), emitted from oculomotoneurons and saccade generator circuits, closely mirrors saccade velocity as a function of time during saccades (for a review, see Cullen, 2024). These brainstem circuits are in turn driven by signals produced primarily in the superior colliculus and frontal eye field. Seminal studies showed that the instantaneous discharge of many neurons in the superior colliculus predict dynamic motor error (Munoz, Pélisson, & Guitton, 1991; Munoz & Wurtz, 1995; Waitzman, Ma, Optican, & Wurtz, 1991). In macaque monkeys performing visual search with a speed–accuracy tradeoff, 66% of a small sample of presaccadic movement neurons in the superior colliculus but only 22% of such neurons in frontal eye field produced discharge rates that scaled significantly with saccade velocity (Reppert et al., 2018b). A recent model of saccade generation offers a mechanism to account for such variation of velocity (Alizadeh & Van Opstal, 2022). The current findings, including the idiosyncrasies, offer new predictions of such models using factorial task designs.

### Considerations for computing saccade vigor

We made several decisions to ensure that the vigor values we calculated were consistent and meaningful. First, we fitted a classic two-parameter exponential function to the peak velocity–amplitude relationship (Bahill et al., 1975; Baloh et al., 1975; Leigh & Zee, 2015). Previous work has also used either a linear fitting (Reppert et al., 2018b) or an exponential fit function with additional parameters (Gibaldi & Sabatini, 2021; Optican & Robinson, 1980). A linear function can be a good approximation for shorter saccades because this relationship is typically linear for saccades of displacement up to ∼20° (Bahill et al., 1975; Collewijn, Erkelens, & Steinman, 1988a). Nonetheless, it risks underestimating vigor for the shortest and longest saccades and overestimating medium-sized saccades. We also fitted the three-parameter exponential function with a horizontal shift term, and the resulting vigor estimates did not significantly differ from those from the two-parameter fit (not reported here), so we retained the more general model. Second, we fitted the exponential function separately to saccades in each direction, because saccades in different directions are generated by different sets of extraocular muscles and the main sequence differs for horizontal, vertical, and oblique saccades (Collewijn et al., 1988a; Collewijn et al., 1988b). Finally, we applied the correction method described by Quaia et al. (2000). This was motivated by the observation that variation in saccade velocity was not fully compensated by the oculomotor system (Figure 2). Given that amplitude and peak velocity covary among saccades to the same target, the main sequence alone cannot account all the variation in the relationship between saccade amplitude and peak velocity. We found that adding the correction method magnified the observed effects.

### Pupil diameter

Pupil size was measured in this study primarily to control for its potential influence on the measurement of saccade metrics. The effects of the two task factors on pupil diameter did not correspond to their effects on vigor or RT. Thus, the vigor effects cannot be attributed to variation in pupil size. Although pupil size diameter varied with task difficulty in both monkeys, these effects were inconsistent across trial types and animals and were small in magnitude. In both monkeys, pupil size was larger on correct than error trials, which may reflect differences in the effort exerted on those trials. However, this study was not designed to examine the effects of decision-related manipulation on pupil size, and features of this task limit what can be inferred from these measurements. First, pupil diameter was sampled at the time of saccade initiation, which varied from trial to trial; therefore, the interval available for pupil to respond to stimuli differed across trials. Second, the colors of the target and distractors varied across trials, and attending to different colored target could affect pupil size. Because both factors can add variance to the pupil measurement, we treat pupil results as descriptive.

To conclude, this study demonstrates that manipulating the quality of decision evidence used for a visual search task influenced both the decision time as well as the saccade dynamics. We separately manipulated the efficiency of two concurrent decision processes—target localization and stimulus–response cue discrimination—and found that these factors can affect RT and saccade vigor differently. This indicates that the processes underlying decision and movement execution are distinct and that manipulations targeting different decision stages can produce dissociable effects on decision time and movement vigor. Together, these findings challenge traditional stochastic accumulator models that assume a sequential and independent relationship between decision and action. Movement vigor offers a novel and valuable constraint on biologically plausible models of decision-making, which can help address the persistent challenge of model mimicry.
